# Overactive bladder – 18 years – Part II

**DOI:** 10.1590/S1677-5538.IBJU.2015.0367

**Published:** 2016

**Authors:** Jose Carlos Truzzi, Cristiano Mendes Gomes, Carlos A. Bezerra, Ivan Mauricio Plata, Jose Campos, Gustavo Luis Garrido, Fernando G. Almeida, Marcio Augusto Averbeck, Alexandre Fornari, Anibal Salazar, Arturo Dell’Oro, Caio Cintra, Carlos Alberto Ricetto Sacomani, Juan Pablo Tapia, Eduardo Brambila, Emilio Miguel Longo, Flavio Trigo Rocha, Francisco Coutinho, Gabriel Favre, José Antonio Garcia, Juan Castaño, Miguel Reyes, Rodrigo Eugenio Leyton, Ruiter Silva Ferreira, Sergio Duran, Vanda López, Ricardo Reges

**Affiliations:** 1 Escola Paulista de Medicina - EPM - Universidade Federal de São Paulo, SP, Brasil; 2 Departamento de Urologia, Universidade de São Paulo, SP, Brasil; 3 Departamento de Urologia, Faculdade de Medicina do ABC, SP, Brasi; 4 Departamento de Urología, Universidad de los Andes, Bogota, Colombia; 5 Departamento de Urología, Escuela Médico Militar, Cidade do México, Mexico; 6 Cátedra de Urologia, Hospital de Clínicas “José de San Martín”, Buenos Aires, Argentina; 7 Departamento de Urologia, Mãe de Deus Center Hospital, Porto Alegre, RS, Brasil; 8 Universidade Federal de Ciências da Saúde de Porto Alegre, Porto Alegre, RS, Brasil; 9 Departamento de Urologia, AC Camargo Hospital, SP, Brasil; 10 Hospital Clinico de la Fuerza Area de Chile, Santiago, Chile; 11 Instituto Mexicano del Seguro Social, Ciudad de Mexico, Mexico; 12 Departamento de Urologia, Hospital Souza Aguiar, RJ, Brasil; 13 Servicio de Urología, del Complejo Médico Policial Churruca Visca, Buenos Aires, Argentina; 14 Centro Policlínico Valencia “La Viña”, Valencia, Venezuela; 15 Hospital Pablo Tobón Uribe, Medellin, Colômbia; 16 Servicio de Urología, Clinica Indisa, Providencia, Chile; 17 Centro de Reabilitação e Readaptação Dr. Henrique Santillo, Goiânia, Brasil; 18 Servicio de Urología, del Hospital Universitario de Caracas, Caracas, Venezuela; 19 Divisão de Urologia, Universidade Federal do Ceará, CE, Brasil

**Keywords:** Overactive Bladder, Muscarinic Antagonists, Beta-adrenergic agonists, Botulinum Toxin, Sacral neuromodulation, Urodynamics

## Abstract

Traditionally, the treatment of overactive bladder syndrome has been based on the use of oral medications with the purpose of reestablishing the detrusor stability. The recent better understanding of the urothelial physiology fostered conceptual changes, and the oral anticholinergics – pillars of the overactive bladder pharmacotherapy – started to be not only recognized for their properties of inhibiting the detrusor contractile activity, but also their action on the bladder afference, and therefore, on the reduction of the symptoms that constitute the syndrome. Beta-adrenergic agonists, which were recently added to the list of drugs for the treatment of overactive bladder, still wait for a definitive positioning – as either a second-line therapy or an adjuvant to oral anticholinergics. Conservative treatment failure, whether due to unsatisfactory results or the presence of adverse side effects, define it as refractory overactive bladder. In this context, the intravesical injection of botulinum toxin type A emerged as an effective option for the existing gap between the primary measures and more complex procedures such as bladder augmentation. Sacral neuromodulation, described three decades ago, had its indication reinforced in this overactive bladder era. Likewise, the electric stimulation of the tibial nerve is now a minimally invasive alternative to treat those with refractory overactive bladder. The results of the systematic literature review on the oral pharmacological treatment and the treatment of refractory overactive bladder gave rise to this second part of the review article *Overactive Bladder – 18 years*, prepared during the 1^st^ Latin-American Consultation on Overactive Bladder.

## Introduction

This second part of the review article *Overactive Bladder (OAB)* – 18 years will address the oral pharmacological treatment and the treatment of refractory overactive bladder. A large number of studies on basic sciences gave rise to conceptual changes in the treatment of OAB. The urothelium – for decades viewed as a simple interface between the detrusor muscle wall and the urinary content – took a critical position in the pathophysiology and therapeutics of OAB. The identification of pharmacologic receptors, interconnected by a complex neurotransmitter network allowed for a better understanding of the role of neural afference in OAB. Oral anticholinergic agents, used by focusing on efference, started to be recognized for their parallel action on the modulation of bladder filling sensations. A new therapeutic option, still with a limited number of studies – beta-adrenergic agonists – were integrated to the armamentarium of oral medications.

Electrical stimulation of the tibial nerve strengthened the physiotherapeutic measures, now focusing on non-responsive cases to primary behavioral approaches and oral medications. Like oral anticholinergic agents, bolutinum toxin type A had its action initially based on the neuromuscular cholinergic blockage and consequent inhibition of the detrusor overactivity. Once again, recent advances on the urothelium physiology led to the recognition of a number of botulinum toxin actions on receptors that have a direct action on the bladder afference. This stimulated the expansion its use from neurogenic cases to OAB patients with or without detrusor overactivity. In parallel, sacral neuromodulation had its indication strengthened for the treatment of refractory OAB, although still in a restricted manner in our community.

Ninety-seven of 2,508 publications previously surveyed in the medical literature for the period from 1997 to 2014 were selected by two independent investigators, as described in the first part of this review. Table 1 outlines the keywords used and the details of the selection of articles. Only full texts in English, Spanish and Portuguese were included. Articles only containing abstracts or published in annals of congresses were excluded. In cases of similar publications, the most recent one and showing the highest level of scientific evidence was selected.

**Table 1:** Method of search of scientific publication at PubMed, Bireme

Groupst	Strategy of search	Filters	Total of identified articles	Total of selected articles
Concept and diagnosis of Overactive bladder	("Urinary Bladder, Overactive/classification"[Mesh] OR "Urinary Bladder, Overactive/diagnosis"[Mesh] OR "Urinary Bladder, Overactive/etiology"[Mesh] OR "Urinary Bladder, Overactive/pathology"[Mesh] OR "Urinary Bladder, Overactive/physiopathology"[Mesh]) OR ("Urinary Incontinence, Urge/classification"[Mesh] OR "Urinary Incontinence, Urge/diagnosis"[Mesh] OR "Urinary Incontinence, Urge/etiology"[Mesh] OR "Urinary Incontinence, Urge/pathology"[Mesh] OR "Urinary Incontinence, Urge/physiopathology"[Mesh])	English/spanish/portuguese + abstract available + 01/01/1985 a 31/05/2014 + humans + >19 years	799	53
Epidemiology	(Urinary Incontinence, Urge or Urinary Bladder, Overactive) and (economics or epidemiology)	English/spanish/portuguese + abstract available + 01/01/1985 a 31/05/2014 + humans + >19 anos	713	51
Conservative Non-Pharmacological Management	(Urinary Incontinence, Urge or Urinary Bladder, Overactive) and (diet therapy or drug therapy or prevention or control or rehabilitation or surgery or treatment or therapy)	English/spanish/portuguese + abstract available + 01/01/1985 a 31/05/2014 + humans + >19 anos Clinical trials, Review, Randomized Clinical Trial	996	35
Pharmacological Management				71
Refractory Overactive Bladder				26

The articles were reviewed and their results compiled during the 1st Latin-American Consultation on Overactive Bladder. The present text was prepared from the works presented in a plenary session of the event with the participation of all members of the Consultation.

### Overactive bladder management with oral medications

Antimuscarinic agents are for many years the first pharmacological treatment option for overactive bladder. Yet, many safety and tolerability-related aspects limit their use (1–4). Special attention should be given to the interaction of antimuscarinic agents with other drugs commonly used by the elderly, prescribed by cardiologists, general practitioners, and psychiatrists. Many of these drugs either have anticholinergic effects or may interfere with the hepatic metabolism and thereby enhance the adverse effects of the antimuscarinic agents that are used in the treatment of overactive bladder.

Five subtypes of muscarinic receptors are found in the human body, classified as M1, M2, M3, M4, and M5. The smooth detrusor muscle and the urothelium mainly contain M2 and M3 receptors. In the urinary tract, while there is a higher proportion of M2 receptors, M3 receptors are responsible for bladder contraction. M2 and M3 receptors are also found in other human organs, such as salivary glands and bowel, which may result in adverse reactions from treatments with antimuscarinic agents (4–7).

Some pharmacokinetic characteristics of the antimuscarinic agents are important in the genesis of side effects, such as the ability to cross the blood-brain barrier determined by the molecular weight and level of liposolubility or lipophilicity (2, 4, 7). In turn, the affinity (pKi) with each receptor also determines differences in the clinical effects. Antimuscarinic agents with lower affinity to M1 receptors (abundant in the central nervous system) provide low risks of neurological adverse effects, even if they cross the blood-brain barrier. In contrast, those with affinity to M1 muscarinic receptors may trigger cognitive disorders, memory loss, drowsiness, confusion, and even accelerate the emergence of dementias. The drug's ability to interact with P glycoproteins, which convey substrates into the nerve cell, also works as a determining factor of adverse effects.

Another important characteristic is the drug exposure time, determined by the metabolism in the liver. It is well known that the elderly loses hepatic mass and blood flow, which may reduce the drug elimination via cytochrome CYP3A4, thereby prolonging the permanence of the drug in the blood stream (7).

Several antimuscarinic agents have been studied in the treatment of overactive bladder, including oxybutynin, tolterodine, solifenacin, darifenacin, trospium, fesoterodine and propiverine (4, 6, 8–11). These drugs mainly have their effects through the inhibition of muscarinic receptors present in the bladder – in both the detrusor muscle and the urothelium (10). In turn, they differ from each other in both their molecular structure and pharmacokinetic proprieties, which leads to differences in their clinical effects (4, 7). The efficacy of the different antimuscarinic agents is very similar, with their main difference lying on their adverse effects.

Several studies having an appropriate methodologic design showed the efficacy of antimuscarinic agents in controlling overactive bladder symptoms. However, adherence to treatment has been an issue, once discontinuation is reported in up to 80% of patients in a one-year follow-up (12). Because overactive bladder is a chronic condition and the long-term treatment with antimuscarinic agents is usually necessary, optimizing the tolerability of the drug is critical in obtaining patient satisfaction. The extended-release (ER) formulations show lower adverse effect rates, probably because they provide more homogeneous plasma concentration levels with lower peaks when compared to immediate-release formulations (13–15).

There are several limiting factors when interpreting and analyzing the results reported by the numerous clinical studies of anticholinergic agents for the treatment of overactive bladder. A relevant factor – reiterated in a number of these studies – is the high frequency of positive response to placebo – approximately 30%, which makes the interpretation of results more difficult. Another factor that stands out is the variability of the study populations in terms of severity of symptoms as well as the assessment methods employed in the clinical trials.

#### Clinical efficacy of antimuscarinic agents in the treatment of overactive bladder

An extensive review of clinical trials evaluating pharmacological therapies of overactive bladder did not reveal any evidence of clinical efficacy differences among the many studied pharmacological agents (16–81). This finding is consistent with the conclusions of many published systematic reviews and meta-analysis (13, 14, 82–93).

From the studies published, a Cochrane review stands out. It demonstrated that solifenacin was superior to tolterodine in the domain of quality of life, according to information provided by patients about their daily urgency and incontinence symptoms. The tolerance to both drugs were similar. Solifenacin showed equivalent efficacy, but with higher tolerance and quality of life than darifenacin (88). The extended-release (ER) formulations provided higher improvement of quality of life than the immediate-release (IR) ones. When compared to tolterodine-IR, oxybutynin-ER had a similar quality-of-life profile, while solifenacin showed superior efficacy and quality of life. In three trials, fesoterodine showed to be more effective, providing superior quality of life when compared to tolterodine-ER (89).

In the SOLIDAR study, the authors compared the selective antimuscarinic agents solifenacin (5 mg) and darifenacin (7.5 mg). Both showed to be equivalent in the results of reducing overactive bladder symptoms; however, solifenacin was superior in quality of life and satisfaction, with a lower incidence of dry mouth (94).

Applying oxybutynin through transdermal patches resulted in similar improvement to that achieved with the oral use of this agent, just with fewer adverse effects (33). Similar results were observed when transdermal oxybutynin and oral tolterodine ER were compared (34).

#### What are the adverse effects of antimuscarinic agents?

Several meta-analyses were designed to evaluate the information provided by clinical trials, specifically regarding adverse effects, which allows us to more easily visualize the safety level of each drug in the distinct presentations and routes of administration.

Chapple et al. (83) conducted a systematic review and meta-analysis of 83 clinical trials in which four formulations were associated with a high risk of discontinuation due to adverse events when compared to placebo – oxybutynin IR, 7.5–10 mg/d; oxybutynin IR, 15 mg/d; propiverine ER, 20 mg/d; and solifenacin, 10 mg/d. Tolterodine ER 4 mg/d was the only formulation associated with low risk of discontinuation when compared to placebo. Reports of dry mouth of any severity were the most commonly seen with all the interventions, with significant difference when compared to placebo. The relative risk ranged from 2.1 to 5.9 and increased when the doses of darifenacin, fesoterodine, solifenacin, and tolterodine were increased. This tendency was not evident for oxybutynin and propiverine. Other adverse effects were significantly high with the active treatment when compared to placebo, including: blurred vision, constipation, erythema, fatigue, pruritus, excessive sweating, and urinary retention.

Another meta-analysis on adverse effects showed fewer patients with dry mouth when using ER formulations of oxybutynin and tolterodine when compared to IR formulation of both drugs, with significant results. There were no within-group differences for the different doses of each medication (82).

Trospium chloride, darifenacin and fesoterodine are active substrates for the P glycoprotein-dependent cell transport system, which means a high reduction of the blood-brain barrier permeability, with low levels in the central nervous system; particularly trospium, which is almost undetectable in the cerebrospinal fluid and a good option in patients with risk of cognitive worsening due to a number of neurological pathologies, such as Parkinson's disease, multiple sclerosis, senile dementia, etc. (95, 96).

A randomized, placebo-controlled, double-blind study compared the effects of oxybutynin topic gel, with oral IR oxybutynin and placebo on the cognitive and psychomotor functions in healthy elder patients (97). When compared to the oral IR formulation, oxybutynin gel has equivalent efficiency, with reduced side effects and a clinically significant effect of less worsening of the cognitive function in healthy older subjects.

Recently, Kessler et al. (98) revealed great methodological and statistic creativity – which they compiled in a systematic review – by using a new meta-analysis referred to as “network meta-analysis.” Through this, after extracting the data from clinical trials, the adverse events were classified into seven categories (gastrointestinal, ocular/visual, urinary tract-related, neurologic, cardiac, respiratory, and dermatological) of the *Common Terminology Criteria for Adverse Events* (CTCAE) and scored using the visual analogic scale (VAS): 0=minimal severity and 10=maximum severity, based on a consensus of ten independent specialists. This meta-analysis studied 60 trials involving 26,229 patients and found a similar global adverse effect profile for darifenacin, fesoterodine, transdermal oxybutynin, propiverine, solifenacin, tolterodine, and trospium, but not for oral oxybutynin when compared to the initial dose. Oxybutynin in doses equal or superior to 10 mg/dL had a worse adverse event profile. Darifenacin, fesoterodine, transdermal oxybutynin, propiverine, and solifenacin showed a significantly better dose-adverse effect correlation. Of all adverse effects, the gastrointestinal ones were the most frequently reported. Only the transdermal oxybutynin at 3.9 mg/dL showed a gastrointestinal profile similar to placebo. Its visual/ocular adverse event profile was similar among the antimuscarinic agents when the initial dose was used. The same was observed for the urinary tract, neurologic, cardiac, and respiratory profiles. While the dermatological adverse effects were minor with the oral administration of the drugs, the adverse event profile was worse with the transdermal administration.

The absolute contraindications for the use of antimuscarinic agents include urinary retention, gastric retention, closed-angle glaucoma, and known hypersensitivity to the pharmacological agent. The relative contraindications include partially obstructed bladder emptying, renal or hepatic changes, excessive use of alcohol, reduced gastrointestinal motility, constipation, and myasthenia Gravis (99).

#### What are the effects of beta-3-adrenergic agonists on overactive bladder symptoms and their adverse effects?

Animal studies and clinical trials have shown that beta-3 agonists relax the detrusor muscle, thereby improving bladder filling and compliance. There is a significant improvement of the incontinence and voiding episodes in 24 hours as well as a significant improvement of the quality of life (100, 101).

The beta-3 agonist Mirabegron was approved for the treatment of overactive bladder in Japan in September 2011 and in Europe and the United States in June 2012 (102). Other beta-3 agonists, such as Solabegron and TRK-380, are still under investigation (103, 104).

Experimental studies have demonstrated that beta-3 adrenergic receptor (β3AR) agonists result in relaxation of the smooth detrusor muscle in humans mediated by the stimulation of the enzyme adenyl cyclase, which leads to the accumulation of cyclic adenosine monophosphate (AMPc). In addition, β3-AR activation seems to inhibit detrusor contraction also due to the release of urothelium-derived inhibitory factor (UDIF) (105, 106).

Seven clinical studies showed that *Mirabegron* (25, 50, and 100 mg) results in a significant reduction of urinary incontinence and voiding episodes in 24 hours (107–111). Five of these studies used 4-mg tolterodine ER as the active control group; five were phase III studies conducted to evaluate safety and efficacy, one was a phase II proof-of-concept study (111), and one was a phase II study of dose determination (109). One of the phase III studies included 1,978 patients from 27 European countries and Australia. All three active groups showed improvement when compared to placebo; however, tolterodine was only superior to placebo at week 4. The main limitation was the short period of 12 weeks of treatment (110).

Another international phase III randomized, placebo-controlled, double-blind, multicenter clinical study conducted in the United States and Canada evaluated the effect of Mirabegron on overactive bladder patients experiencing symptoms for over three months. When compared to placebo, 50 mg and 100 mg Mirabegron resulted in a superior reduction of the number of incontinence and voiding episodes in 24 hours (112).

One study evaluated the urodynamic impact of Mirabegron on bladder emptying in men. In this study, 200 men with lower urinary tract symptoms associated with benign prostatic hyperplasia were randomized to receive 50 mg or 100 mg of Mirabegron or placebo daily during 12 weeks. There was no significant difference between groups, in terms of urinary flow and the detrusor pressure on the maximum flow (100).

The pooled analysis of three phase III studies revealed that the global incidence of adverse events and serious adverse events was similar among the groups treated with Mirabegron, tolterodine or placebo (15, 113).

One study evaluated the potential of Mirabegron to cause a change in the cardiac repolarization in healthy patients. The authors observed that Mirabegron did not prolong the QTc interval at the doses of 50 or 100 mg; only the 200 mg dose of Mirabegron caused an increase of the QTc interval superior to 10 ms in women (114).

Mirabegron dose is recommended to be reduced in patients with renal and hepatic impairment, and because it inhibits CYP enzyme, caution should be taken in patients using digoxin and metoprolol (101).

### Treatment of refractory overactive bladder

Patients that are refractory to conservative treatment, including behavioral, physiotherapeutic and pharmacological treatments should be evaluated by a specialist, in case they want additional treatment.

Patients considered refractory are those failing an adequate behavioral treatment, for long enough to evaluate the results, usually three months, and that have also failed the pharmacological treatment with at least one antimuscarinic agent, taken for at least 4–8 weeks. Pharmacological treatment failure may include lack of efficacy, drug intolerance, or absolute contraindication to its use.

Treatment options after failure of conservative treatment pose significant risks to patients. Therefore, before indicating such treatments, a thorough evaluation should take place in order to ensure that the complaints are truly resulting from idiopathic overactive bladder, rather than secondary to other clinical conditions.

The therapeutic options after conservative treatment failure include electrical stimulation of the tibial nerve, botulinum toxin injection into detrusor, and sacral neuromodulation (SNM). Each of these alternatives has advantages and disadvantages that will be addressed in the respective topic.

The use of these therapeutic options requires a careful selection of patients and detailed education. Such treatments may be offered in any order; that is, none of them could be considered as being superior to the others. If a patient fail in one of these treatments, he or she can be a candidate to another one. The efficacy of a combination of these therapeutic modalities is unknown.

#### Electrical stimulation of the tibial nerve

The peripheral electrical stimulation of the tibial nerve has been used in the treatment of bladder filling and urgency urinary incontinence symptoms. The impact of the treatment depends on the severity of the symptoms before treatment, the number of weekly sessions, and the evaluation time point following the procedure. This technique can be performed transcutaneously or percutaneously (115). The electrical stimulation has its effect by inhibiting the detrusor activity via afferent action of the pudendum nerve (116). In order to stimulate the tibial nerve percutaneously (PTNS), a 34G needle is inserted in a cephalic position in relation to the internal malleolus and for electrical stimulation, a low voltage (9V) with a width of 0–10mA is used, with a fixed frequency of 20Hz and pulse width of 200 ms. The majority of the studies evaluating this therapeutic modality is of case sequences, without a control group, and exclusively conducted in women. The equipment, as well as the electrical stimulation parameters and weekly session protocols in these studies greatly varied. Most included relatively small samples (14–60 patients) with a short follow-up (12 weeks). In comparative studies of the use of PTNS with placebo, a reduction of more than 50% of the urgency and urge-incontinence symptoms in 54–71% of patients receiving against 0–22% of patients in the control group (117–119). Most studies showed improvement of quality of life as evaluated by different questionnaires (118–120). Systematic reviews demonstrated that 37–100% of patients treated with PTNS experienced treatment success, with different evaluation criteria having been adopted and minimum adverse effects (121–123). A recent meta-analysis indicated a seven-fold higher chance of clinical improvement in the PTNS treated group versus control group.

Studies evaluating the duration of PTNS effects without further treatments showed, after completion of the initial protocol, maintenance of efficacy for up to six months (124, 125). Studies with up to three-year follow-ups demonstrated sustained efficacy upon a monthly treatment, after one cycle for 12 weeks (117, 126). Maintenance protocols are variable and tend to be individualized. Typically, after the 12 weeks of initial treatment, the interval between sessions is progressively increase until it reaches one session a month (118, 126). Patients experiencing recurrence with this maintenance protocol are adjusted and receive reapplications within shorter intervals. In the SUmiT study, 41% of patients required less than one session a month, 55% needed 1–2 monthly sessions, and 4% more than two sessions a month. The most commonly seen adverse effects of the treatment with PTNS include pain at the puncture site (medial malleolus), local bleeding, paresthesia, and excoriations. These effects are rare and affect about 5% of patients (118, 120). It is important to stress the high rate of abandon at the long term follow-up, which reaches as much as 42% (118).

#### Botulinum toxin

The botulinum toxin type A counting with the largest number of scientific studies and that is approved for the treatment of overactive bladder by the *Food and Drug Administration* (FDA) and *European Medicines Agency* (EMA) is the one called onabotulinumtoxinA. Other botulinum toxins, such as abobotulinumtoxinA and incobotulinumtoxinA, were less frequently studied for bladder indication. Their efficacy and safety are not well known. In addition, their use is not provided in label for this indication. For this reason, our text will be based on the data published on onabotulinumtoxinA.

The treatment with onabotulinumtoxinA can be used to patients that are refractory to the conservative treatment, after detailed education. Patients must be able to attend frequent follow-up visits in order to evaluate the bladder emptying pattern. They must accept the possibility of intermittent bladder catheterization, if necessary.

The efficacy and side effects of the treatment with onabotulinumtoxinA depend on the dose used. The recommended dose for most patients should be 100 or 150 U. The use of 150 U results in a discrete improvement of efficacy, but higher risk of urinary retention (127–130).

Most studies evaluated the bolutinum toxin injection into the body of the bladder, sparing the bladder trigone (127–131). Different protocols were used, including injections into 10–20 bladder sites and dilution in such a way to inject 0.5–1.0 mL per site. OnabotulinumtoxinA injection should be performed deeply into the detrusor. Submucosal injection was less often evaluated and its results are not well known.

OnabotulinumtoxinA injection can be performed with local anesthesia, sedation, spinal block, or general anesthesia. The procedure with local anesthesia usually requires the use of flexible cystoscope and a longer injection needle, at a higher cost. With other forms of anesthesia, the procedure can be performed with the aid of rigid cystoscope and a shorter injection needle. This equipment is cheaper and easily available. The choice of the type of anesthesia should be agreed upon by both physician and patient, considering the equipment availability.

OnabotulinumtoxinA at a dose of 100 or 150 U results in improvement of several clinical parameters and the quality of life. The impact of the treatment on these parameters depends on the severity of symptoms before treatment, the dose used, and the evaluation time point after the procedure. In comparative studies of onabotulinumtoxinA with placebo, a reduction of more than 50% of urgency and urge-incontinence symptoms was observed in 57–68% of patients receiving 100 U versus 27–30% of those receiving placebo. Total continence was observed in 23–55% of patients receiving 100 U versus 6–11% of those receiving placebo (129–131). The improvement of the quality of life, as evaluated through different questionnaires, was 60–63% among patients receiving 100 U versus 27–29% of patients receiving placebo (130, 131). The urodynamic efficacy of the treatment with onabotulinumtoxinA has been little studied. Some studies showed improvement of the cystometric bladder capacity (25%) and absence of the detrusor overactivity (35–39%) (132–134).

The most commonly seen adverse effects following onabotulinumtoxinA injection include urinary tract infections, increase of the post-void residual volume, and urinary retention requiring bladder catheterization (127, 129–131). Urinary tract infections occurred in 15–55% of patients receiving 100 U versus 6–28% of those receiving placebo. Post-void residue superior to 150 mL was observed in 7–26% of patients receiving 100 U versus up to 1% of those receiving placebo (127–131). Urinary retention requiring bladder catheterization was observed in 3–18% of patients receiving 100 U versus 0–2% of those receiving placebo (127–131, 134). The urinary retention may last a few days to several weeks, and in less than 50% of patients it lasts more than six weeks (128, 130). Other side effects include hematuria (4–18%) and muscular weakness (3–9%).

The majority of studies evaluated patients during three months, and some studies evaluated patients for 6–12 months, with sustained efficacy throughout this period (127–131, 134, 135).

Some factors are associated with a higher chance of complications, particularly urinary retention requiring bladder catheterization. Among these factors, being male, over 75 years old, and initial post-void residue superior to 100 mL (132).

Most of the studies evaluating reinjections are retrospective and without a control group. The interval between injections varied from 6 to 14 months (136–139), with most of them showing maintenance of the treatment effects after reinjections (136, 137, 140).

Treatment discontinuation is dependent on factors such as follow-up time, treatment efficacy and side effects, particularly urinary retention and urinary tract infections. In a study following up patients for 60 months, the treatment discontinuation rate was 64% mainly due to the emergence of complications such as urinary retention and urinary tract infections and for the lack of efficacy of the initial injection (138).

The treatment with onabotulinumtoxinA may improve patients with moderate-to-severe symptoms of overactive bladder that did not respond to the conservative treatment. The dose of 100 U results in a lower rate of side effects. The benefits of the treatment with onabotulinumtoxinA at a dose of 100 U outweigh the risks and discomforts in patients with refractory overactive bladder to the conservative treatment. It is critical that patients that are candidates for this treatment are carefully educated about the risks and they should accept the possibility that they need bladder catheterization for relief. Patients should also be informed that the effects of the treatment diminish over time and the vast majority will need reinjections. The current evidence limitations include short follow-up time in better designed studies and variations of protocol injections and adverse effect reporting.

#### Sacral neuromodulation

Treatment with sacral neuromodulation (SNM) can be offered to patients with overactive bladder symptoms that are refractory to the conservative treatment after detailed education.

Studies evaluating this therapeutic modality are based on the use of the equipment Interstim (Medtronic, Minneapolis, USA), which has gone through changes in the last years, with the incorporation of a minimally invasive surgical technique, lower volume battery, anchored electrodes, and the introduction of the percutaneous stimulation testing (percutaneous nerve evaluation – PNE).

The treatment with SNM involves a test phase (first phase), which may be performed with the implantation of a temporary electrode (PNE) or the implantation of the permanent electrode. The implantation of the electrode for PNE is simpler and less expensive; however, the results are inferior to those achieved with the permanent electrode. Studies reported as high success rates during the test phase with PNE as 44–60% (141–144) and 69–81% with the implantation of the permanent electrode (141, 142, 145, 146). Around 44% of patients failing the PNE test have positive response when undergoing a new test with the implantation of the permanent electrode (142).

The second phase of the treatment is indicated in cases showing good clinical response to the test phase. In general, a 50% or more improvement of urgency urinary incontinence symptoms is the parameter used as the success criterion. This treatment phase consists of the implantation of the generator in cases where the permanent electrode was implanted during the test phase. In cases where the first phase was performed with PNE, the second phase consists of the implantation of the entire system, including the permanent electrode and the generator.

Most of the studies evaluating this treatment modality consist of observational series without a control group. Some studies report long-term results (up to five years). Overall, studies show significant improvement of several clinical and quality-of-life parameters.

Improvement of more than 50% of urgency and incontinence symptoms is observed in 65–87% of patients in the short-term follow-up (up to six months) (147). Most of the patients have good results in the long run. Improvement of more than 50% of urgency and urgency incontinence symptoms is observed in 62–70% of patients after a five-year follow-up (147–151). The satisfaction rates of patients followed up for more than five years is as high as 60–80% (152). The healing of urgency urinary incontinence is observed in 20–55% of patients (151, 153).

Treatment results may be long-lasting, although the adverse effect and additional surgery rates are significant. However, the potential variability of the rates observed in old studies as well as in more recent ones incorporating technological advances should be taken into account. The most commonly reported adverse effects include local pain at the generator implantation site (3–20%), pain at the electrode site (4–19%), electrode migration (1–9%), infection (2–14%), shock sensation (5–8%), and need for revision surgery (6–39%). In most studies, the revision surgery rates were superior to 30%. There is evidence that the current procedures, with less invasive surgical technique and the use of anchored electrodes, result in lower adverse effect rates (147–151, 154, 155). In a study evaluating patients from Medicare undergoing treatment with SNM in the period from 1997 to 2007, the generator removal rate was about 11% in a 60-month follow-up on average (156).

Patients should be informed about the need for periodic replacement of the generator, with the interval depending on the stimulation parameters used. Patients should be able to handle the remote control in order to optimize the device functioning. Patients must accept the fact that magnetic resonance imaging is contraindicated for individuals using the device (except for cranial magnetic resonance imaging, provided that the manufacturer's instructions are followed). By taking into consideration the negative effects of the overactive bladder symptoms on patients' quality of life, it is possible to state that the benefits from SNM outweigh the risks and discomforts of the treatment in selected, carefully educated patients.

Recommendation of this treatment is limited due to the fact that the majority of studies have an observational design, small samples, different studies reporting results in the same group of patients, and the lack of information on the protocols used by patients in order to maintain the treatment results in the long term.

#### Surgical treatment of overactive bladder

In rare occasions, bladder augmentation or urinary diversion may be considered in highly selected patients with refractory overactive bladder. Almost all papers reporting results from bladder augmentation are based on populations with neurogenic bladder dysfunctions. Very little is known about its use in patients with idiopathic overactive bladder. The risks of the procedure are high and many patients may need intermittent catheterization for bladder emptying.
